# Hashimoto’s Thyroiditis and Autoimmune Gastritis

**DOI:** 10.3389/fendo.2017.00092

**Published:** 2017-04-26

**Authors:** Miriam Cellini, Maria Giulia Santaguida, Camilla Virili, Silvia Capriello, Nunzia Brusca, Lucilla Gargano, Marco Centanni

**Affiliations:** ^1^Endocrinology Unit, Department of Medico-Surgical Sciences and Biotechnologies, Sapienza University, Latina, Italy

**Keywords:** thyroiditis, polyglandular autoimmune syndrome, thyroxine malabsorption, gastric atrophy, pernicious anemia, *Helicobacter pylori* infection, cellular immunity

## Abstract

The term “thyrogastric syndrome” defines the association between autoimmune thyroid disease and chronic autoimmune gastritis (CAG), and it was first described in the early 1960s. More recently, this association has been included in polyglandular autoimmune syndrome type IIIb, in which autoimmune thyroiditis represents the pivotal disorder. Hashimoto’s thyroiditis (HT) is the most frequent autoimmune disease, and it has been reported to be associated with gastric disorders in 10–40% of patients while about 40% of patients with autoimmune gastritis also present HT. Some intriguing similarities have been described about the pathogenic mechanism of these two disorders, involving a complex interaction among genetic, embryological, immunologic, and environmental factors. CAG is characterized by a partial or total disappearance of parietal cells implying the impairment of both hydrochloric acid and intrinsic factor production. The clinical outcome of this gastric damage is the occurrence of a hypochlorhydric-dependent iron-deficient anemia, followed by pernicious anemia concomitant with the progression to a severe gastric atrophy. Malabsorption of levothyroxine may occur as well. We have briefly summarized in this minireview the most recent achievements on this peculiar association of diseases that, in the last years, have been increasingly diagnosed.

## Introduction

The thyrogastric syndrome was initially described in the early 1960s and initially characterized by the presence of thyroid autoantibodies in patients with pernicious anemia, the latter being used as synonymous for atrophic gastritis (1). More recently, the autoimmune gastritis has been better characterized classifying chronic atrophic gastritis, with or without the PA, based on the histological evaluation and the presence of serum parietal cell (PCA) and/or intrinsic factor (IFA) autoantibodies (2, 3). Based on these criteria, the association between autoimmune thyroid disorders and chronic autoimmune gastritis (CAG) has also been reassessed (4, 5) and nowadays is included in the adult form of polyglandular autoimmune syndrome (PAS), characterized by two or more endocrine and non-endocrine autoimmune disorders (6). In particular, Betterle and colleagues have proposed the inclusion of thyrogastric syndrome in the PAS Type 3b, in which Hashimoto’s thyroiditis (HT) occurs also associated with non-endocrine autoimmune gastrointestinal disorders and where it plays a pivotal role (7, 8). This is in keeping with the evidence that chronic autoimmune thyroiditis represents the more prevalent autoimmune disorder worldwide making the frequency of thyrogastric syndrome quite high (4). This notion is supported by the high percentage (12–40%) of positivity of PCA in adult patients with HT (9) which, in turn, is present in approximately 40% of patients with atrophic gastritis (10). Besides the fact that the thyroid and the stomach share some embryological and biochemical features (11), some intriguing similarities have been observed even in the putative pathogenic mechanisms, which characterize the thyrogastric syndrome (12). Furthermore, some specific clinical features characterize or lead to the suspicion of the coexistence of both thyroid and gastric autoimmune (13, 14) disorders. These similar peculiar features will be briefly described in this minireview.

## Thyroid and Stomach: Embryologic Derivation and Role of Na^+^/I^−^ Symporter

The thyroid gland and stomach, despite the different localization and function, share some similar morphologic and functional characteristics, likely due to their common embryologic origin (11). In fact, the thyroid gland develops from the primitive gut and therefore thyroid follicular share with parietal cells the same endodermal origin. Also, both these cells are polarized and are characterized by the presence of apical microvilli housing enzymatic activities.

Furthermore, gastric mucosal and thyroid follicular cells both show the ability to concentrate and transport iodine across the cell membrane (15). This process is mediated by the Na^+^/I^−^ symporter (15) and involves similar enzymes with an efficient peroxidase activity (12) (Table 1). Furthermore, besides its essential role for the synthesis of thyroid hormones, iodine regulates the proliferation of gastric mucosal cells (16). In fact, in the presence of gastric peroxidase, iodine acts as an electron donor and participates in the removal of free oxygen radicals, thus playing an antioxidant action (17). These effects may explain the regulatory role of iodine in the proliferation of mucosal cells and its protective role against gastric carcinogenesis (11, 16). This hypothesis has been confirmed by the reported link among iodine deficiency, goiter, and increased risk of developing gastric cancer (18).

**Table 1:** **Shared characteristics between thyroid and stomach**.

Embryological origin	Primitive gut for both thyroid and stomach
Cell features	Presence of cells polarity Cells characterized by apical microvilli
Biochemical features	Presence of Na^+^/I^−^ symporter (sodium-iodide symporter) Presence of peroxidase isoenzymes (TPO and GPO)
Function	Ability to concentrate iodine Presence of antioxidative activities Secretion of mucinous glycoproteins: thyroglobulin and mucine
Pathogenesis	Cellular immune involvement Similarity of autoaggressive processes Mechanisms of cellular damage Expression of autoantigens and related cross-reacting autoantibodies
Pathology	Clinically related autoimmune disorders Peculiar associative clinical features

## Chronic Autoimmune/Hashimoto’s Thyroiditis and CAG

Chronic lymphocytic thyroiditis is the most frequent autoimmune disorder and represents the prototype of organ-specific autoimmunity (19). Its prevalence, despite some difference of sex, age, race, and iodine intake, reaches about 5% in the general population (20). Much less frequent is the chronic autoimmune atrophic gastritis (type A gastritis or body/fundus gastritis), which represents only some 5% of the whole spectrum of chronic gastritis and must be differentiated from the one associated with chronic *Helicobacter pylori* (Hp) infection (type B gastritis or antral gastritis) (21, 22). HT is characterized by diffuse inflammatory changes with lymphocytic infiltration of the thyroid gland, leading to the destruction of the thyroid epithelial cells with subsequent fibrosis (23). Similarly, autoimmune gastritis is a chronic inflammatory disease involving gastric body and fundus, with the progressive reduction and/or disappearance of the native gastric glands that are sometimes replaced by intestinal or pyloric epithelium (metaplasia) (3). The natural history of HT is the progressive reduction of thyroid function till overt hypothyroidism (24) with a rate of progression of 2–4% per year (23), while that of gastric atrophy features the progressive reduction, till disappearance, of parietal cells, leading to reduced or absent acid production (3, 22). These alterations interfere with absorption of essential nutrients leading, at first, to iron-deficient anemia, followed by PA if the self-injurious process involves the IFA (13). Increased risk of developing neuroendocrine tumors and gastric adenocarcinoma is also associated with the severity of damage of gastric mucosa (22).

### Pathogenesis

Both these autoimmune disorders are characterized by a complex interaction between genetic susceptibility and environmental factors that results in the loss of immune tolerance to self-antigens and in the development of autoimmune diseases. The loss of immune tolerance may involve alteration both in the central tolerance with reactive T cells escaped from intrathymic deletion and in the peripheral tolerance as in the case of defective T regulatory lymphocytes (25, 26). Genetic susceptibility has been confirmed for both diseases since their incidence is higher among identical twins and first-degree relatives as well as their presence may be observed in association with further autoimmune disorders (6, 7, 20, 26). Both of these disorders show a definite association with different HLA aplotypes; in HT, it has also been proven that the involvement of many other immunoregulatory genes (27), while this issue has not been elucidated in the pathogenesis of human autoimmune gastritis (26).

Several environmental factors seem to be involved in the pathogenesis of HT (excessive iodine intake, selenium deficiency, and specific drugs use), while very weak evidence supports a role for infectious agents as trigger for this disease (hepatitis C virus, HHV-6, and Yersinia) (27). The role of environmental factors in triggering autoimmune gastritis has been more studied and a stronger link between *H. pylori* infection and CAG has been detected, despite not sufficient to establish a causative relationship between these two diseases (21). *H. pylori* infection affects approximately 50% of the world population and is in turn the most common cause of chronic gastritis. At first, the *H. pylori* infection involves the gastric antrum, but in some patients it may extend into the gastric body (pangastritis) and, in genetically predisposed individuals, it may be a trigger for autoimmune atrophic gastritis, being this hypothesis still debated (3, 28, 29). The pathogenic link may be found in a cross-reactivity mechanism (molecular mimicry) (30): in fact, the Hp infection may induce the proliferation of CD4^+^ T lymphocytes that recognize epitopes of *H. pylori* structurally similar to those of H^+^/K^+^ATPase, an enzyme found on the apical membrane of parietal cells (31). Indeed, dendritic cells may present these shared epitopes to naïve T cells and, in the absence of peripheral tolerance, a Th1-driven autoreactive clone is activated (28). Again, the cellular immune mechanisms of autoimmune thyroiditis show some similarities with those of CAG. In HT, inflammation leads to secretion of IFN-γ, a cytokine turning thyrocytes into antigen-presenting cells (32). The variation of costimulatory factors that drive the binding between an autoantigen and the T-cell receptor allows the proliferation and polarization of autoreactive effector lymphocytes (27). Due to a Th17 cell polarization, the inflammatory process and the subsequent fibrosis seem to prevail in the early phase of thyroiditis (33); in a later phase, when the lymphocytic infiltration and the parenchymal destruction are prevalent, a polarized Th1 profile has been reported (34, 35). The Th1 lymphocytes are able to aid cytotoxic T-lymphocytes and to produce specific cytokines (TNF-α and IFN-γ) able to induce the cellular apoptosis (35) in thyroid cells. The association of a gastric autoimmune disorder has been shown to add a Th2 cytokine profile to the described ones (36). The precise mechanism leading to thyrocytes and/or parietal cell death is still unknown. However, the involvement of Fas upregulation in thyrocytes, due to IL-1beta produced by activated macrophages, has been proven (37). Normal thyrocytes, in fact, express FasL but not Fas, while their concomitant expression induces an autocrine interaction that may represent the main mechanism inducing apoptosis (37). In experimental autoimmune gastritis, also parietal cells express Fas that, in this case, could trigger apoptosis by binding Fas-ligand on infiltrating T cells (28). Following cells damage, the production of specific autoantibodies ensues in epiphenomenal fashion (34). Cellular and humoral immune cooperation characterizes both autoimmune thyroiditis and gastritis leading to the production of specific autoantibodies (antithyroperoxidase, antithyroglobulin, and antiparietal cell antibodies). These autoantibodies are of paramount importance in the diagnosis but of little, if any, in the pathogenesis of these autoimmune disorders.

## Clinical Aspects of Thyrogastric Syndrome

Clinical pathological aspects of this association are attributable to malabsorption of iron and thyroxine, both linked to a reduced gastric acid secretion.

### Iron Deficiency and PA

Chronic atrophic gastritis is clinically silent in most cases and only a small percentage of patients may complain about dyspeptic symptoms. A well-described clinical feature of thyrogastric syndrome is represented by the presence of an iron-deficient and/or a PA. In fact, it has been demonstrated that an iron-deficient anemia, refractory to oral iron therapy, in patients with HT, may be due to chronic atrophic gastritis (13). The clinical signs of this disease appear after several years of its onset, when the progressive reduction to disappearance of the parietal cells leads to atrophy of the gastric mucosa, impairing the absorption of iron, vitamin B12 (cobalamin), folate, and other nutrients (22). At the physiologic acid pH (1.5–2) of the stomach, ascorbic acid, the most active form of vitamin C, allows iron reduction from the nutritional ferric (Fe +++) to the ferrous form (Fe ++), thus forming a complex that drives the absorption in the upper portion of the small intestine (22). In the initial phase of the atrophic gastritis, the damage of parietal cells can lead to iron deficiency microcytic anemia as the only clinical sign (38). When the gastric atrophy becomes severe and/or the IFA is no longer produced, even the absorption of cobalamin becomes compromised. Besides hydrochloric acid that promotes the separation of vitamin B12 from food, the parietal cells also produce the IFA that binds cobalamin and pipes it to the distal ileum, where it is absorbed following a binding to specific receptors (39). Vitamin B12 deficiency is responsible for hematologic changes (macrocytic anemia) and specific neurological disorders (paresthesia and neuritis) which are peculiar of PA (22).

### Thyroxine Malabsorption in Chronic Gastritis

The worldwide used pharmaceutical form of thyroxine (sodium levothyroxine, T_4_) is obtained by native hormone through its salification with sodium hydroxide. The absorption of T_4_ occurs in all sections of small intestine being anyway incomplete and ranging from 62 to 82% of the ingested dose (40). However, increasing evidence of a relevant role of the intact gastric acid secretion on the subsequent intestinal absorption of sodium levothyroxine has been reported in the last years (41). In fact, an increased therapeutic T4 dose has been described in patients with gastric disorders (Hp infection, chronic gastritis, gastric atrophy) or chronically treated with proton pump inhibitors or in non-fasting patients (41–43). All these conditions are characterized by a modified gastric pH that may affect T4 absorption by changing the ionization status, as already described for iron, or the dissolution process of the pharmaceutical T4 form. Furthermore, *in vitro* studies have shown the pH dependency of the dissolution profile of different T4 preparations (44). This evidence boosted the research for novel thyroxine formulations as liquid or softgel capsules. These ones showed, as compared to the classic tablet formulation, a similar or better bioavailability as well as a lower number of excipients (45, 46). In clinical studies, softgel or liquid formulations performed better in patients with gastric disorders (47, 48) and in proton pump inhibitors users (49, 50).

In conclusion, the association of thyroid and gastric autoimmune disorders represents a frequent syndrome, included in the autoimmune polyendocrine syndrome. The similar or even common biochemical and pathogenic features fully support the term thyrogastric disease described some 60 years ago. From a clinical standpoint, the presence of iron-deficient anemia and thyroxine malabsorption may represent an alert signal for the presence of a gastric disorder in patients with thyroid autoimmunity and should trigger a specific diagnostic workup.

## Author Contributions

All authors listed have made substantial, direct, and intellectual contribution to the work and approved it for publication.

## Conflict of Interest Statement

The authors declare that the research was conducted in the absence of any commercial or financial relationships that could be construed as a potential conflict of interest.
